# Automatic detection of actionable radiology reports using bidirectional encoder representations from transformers

**DOI:** 10.1186/s12911-021-01623-6

**Published:** 2021-09-11

**Authors:** Yuta Nakamura, Shouhei Hanaoka, Yukihiro Nomura, Takahiro Nakao, Soichiro Miki, Takeyuki Watadani, Takeharu Yoshikawa, Naoto Hayashi, Osamu Abe

**Affiliations:** 1 Division of Radiology and Biomedical Engineering, Graduate School of Medicine, The University of Tokyo, 7-3-1 Hongo, Bunkyo-ku, Tokyo, 113-8655 Japan; 2 The Department of Radiology, The University of Tokyo Hospital, 7-3-1 Hongo, Bunkyo-ku, Tokyo, 113-8655 Japan; 3 The Department of Computational Diagnostic Radiology and Preventive Medicine, The University of Tokyo Hospital, 7-3-1 Hongo, Bunkyo-ku, Tokyo, 113-8655 Japan

**Keywords:** Radiology reports, Actionable finding, Natural language processing (NLP), Bidirectional encoder representations from transformers (BERT), Deep learning

## Abstract

**Background:**

It is essential for radiologists to communicate actionable findings to the referring clinicians reliably. Natural language processing (NLP) has been shown to help identify free-text radiology reports including actionable findings. However, the application of recent deep learning techniques to radiology reports, which can improve the detection performance, has not been thoroughly examined. Moreover, free-text that clinicians input in the ordering form (order information) has seldom been used to identify actionable reports. This study aims to evaluate the benefits of two new approaches: (1) bidirectional encoder representations from transformers (BERT), a recent deep learning architecture in NLP, and (2) using order information in addition to radiology reports.

**Methods:**

We performed a binary classification to distinguish actionable reports (i.e., radiology reports tagged as *actionable* in actual radiological practice) from non-actionable ones (those without an *actionable* tag). 90,923 Japanese radiology reports in our hospital were used, of which 788 (0.87%) were actionable. We evaluated four methods, statistical machine learning with logistic regression (LR) and with gradient boosting decision tree (GBDT), and deep learning with a bidirectional long short-term memory (LSTM) model and a publicly available Japanese BERT model. Each method was used with two different inputs, radiology reports alone and pairs of order information and radiology reports. Thus, eight experiments were conducted to examine the performance.

**Results:**

Without order information, BERT achieved the highest area under the precision-recall curve (AUPRC) of 0.5138, which showed a statistically significant improvement over LR, GBDT, and LSTM, and the highest area under the receiver operating characteristic curve (AUROC) of 0.9516. Simply coupling the order information with the radiology reports slightly increased the AUPRC of BERT but did not lead to a statistically significant improvement. This may be due to the complexity of clinical decisions made by radiologists.

**Conclusions:**

BERT was assumed to be useful to detect actionable reports. More sophisticated methods are required to use order information effectively.

## Background

A radiology report may include an actionable finding that is critical if left overlooked by the referring clinician [1]. However, clinicians can fail to see mentions of actionable findings in radiology reports for various reasons, and such failure in communication can delay further procedures and impact the prognosis of the patient [2]. Therefore, fast and reliable communication on actionable findings is essential in clinical practice.

Information technologies are helpful in identifying and tracking actionable findings in radiology reports [3, 4]. Handling such information in radiology reports seems a difficult task because radiology reports usually remain unstructured free texts [5]. However, thanks to recently developed natural language processing (NLP) technologies, the detection of radiology reports with actionable findings has been achieved, as well as various other tasks using radiology reports [6]. The aim of this study is to automatically detect reports with actionable findings by NLP-technology-based methods.

Many researchers in previous studies have used NLP technologies to automatically detect specific findings or diseases in radiology reports. Some of them stated that their goal is to assist in tracking and surveillance of actionable findings, the details of which are summarized in Table 1 [7–26]. Some studies in Table 1 have the following features: (1) Multiple or all types of pathological entities are covered [7–15]. (2) The ground truth is based on clinical decisions, not just on the existence of specific expressions in radiology reports [16–18]. These two features can both lead to comprehensive detection of radiology reports with actionable findings. However, there have been no studies that use both features to the best of our knowledge.

**Table 1:** Summary of previous studies of automatic detection of radiology reports with actionable findings, along with this study

	Target language	Multiple diseases	Use of labels in clinical practice	Criteria for positive class	Target sections in radiology reports	Methods
Meng et al. [7]	English	Yes	No	Expressions suggesting the need to promptly communicate to the referring clinician	Impression	Existing tool
Helibrun et al. [8]	English	Yes	No	Expressions suggesting specific critical findings	Impression	Existing tool
Carrodeguas et al. [9]	English	Yes	No	Follow-up recommendations	Impression	SML, LSTM
Yetisgen-Yildiz et al. [10]	English	Yes	No	Follow-up recommendations	Order information, findings, impression	SML
Yetisgen-Yildiz et al. [11]	English	Yes	No	Follow-up recommendations	Order information, findings, impression	SML
Dutta et al. [12]	English	Yes	No	Follow-up recommendations	Findings, impression, recommendation	Existing tool
Lau et al. [13]	English	Yes	No	Follow-up recommendations	(Not specified)	GRU
Dang et al. [14]	English	Yes	No	Follow-up recommendations	(Not specified)	Decision tree
Imai et al. [15]	Japanese	Yes	No	Expressions suggesting malignancy	Findings	Syntactic analysis
Lou et al. [16]	English	No	Yes	Reports pointing at indeterminate or suspicious upper abdominal mass	(Not specified)	SML
Danforth et al. [17]	English	No	Yes	ICD-9 codes suggesting lung nodules	(Not specified)	Rule base
Garla et al. [18]	English	No	Yes	Expressions suggesting potentially malignant liver lesions	(Not specified)	SML
Farjah et al. [19]	English	No	No	Expressions suggesting lung nodules	(Not specified)	Existing tool
Gershanik et al. [20]	English	No	No	Expressions suggesting lung nodules	Findings, impression	Existing tool
Oliveira et al. [21]	English	No	No	Expressions suggesting incidental lung nodules	Order information, findings	Rule base
Pham et al. [22]	French	No	No	Expressions suggesting incidentalomas	Order information, findings, impression	SML
Mabotuwana et al. [23]	English	No	No	Follow-up recommendations	(Not specified)	Rule base
Morioka et al. [24]	English	No	No	Expressions suggesting abdominal aorta aneurysm	(Not specified)	Existing tool
Xu et al. [25]	English	(Not specified)	No	Follow-up recommendations	Order information, findings, impression	SML
Fu et al. [26]	English	No	No	Expressions suggesting silent brain infarction or white matter disease	(Not specified)	Rule base, SML, CNN
This study	Japanese	Yes	Yes	Reports with an *actionable* tag	Order information, findings, impression	SML, LSTM, BERT

BERT = Bidirectional Encoder Representations from Transformers, CNN = Convolutional Neural Network, GRU = Gated Recurrent Units, LSTM = Long Short-Term Memory, and SML = Statistical Machine Learning

In our hospital, for better communication and tracking of any actionable findings, an *actionable* tagging function was implemented in our radiological reporting system and this function has been in operation since September 9, 2019. Thus, adopting *actionable* tags for labeling can provide a dataset based on clinical decisions for all types of pathological entities.

In addition to the free texts in radiology reports, the free texts that are input in the ordering form by the referring clinician (hereafter, order information) may also be useful for detecting radiology reports with actionable findings. That is, if serious and incidental findings are present, some gaps can be found between the order information and the radiology report.

Several research groups have investigated the automatic detection of actionable findings based on statistical machine learning [9–11, 16, 18, 22, 25, 26]. However, these methods are mainly based on the frequency of words in each document, and other rich features such as word order and context are hardly taken into account. Recently, bidirectional encoder representations from transformers (BERT), one of the Transformer networks [27, 28], has attracted much attention because it achieves state-of-the-art performance in various NLP tasks. For better detection of radiology reports with actionable findings, BERT is worth using for two reasons: (1) BERT can use linguistic knowledge not only from an in-house dataset but also from a corpus (a set of documents) for pre-training [29]. (2) BERT is able to capture the relationship between two documents [28], which may enable it to perform well for a pair comprising order information and a radiology report. BERT has been used in several very recent studies of classification tasks in radiology reports [30, 31]. To the best of our knowledge, however, there have been no attempt to use BERT for the automated detection of radiology reports with actionable findings.

In this study, we investigate the automated detection of radiology reports with actionable findings using BERT.

The contributions of this study are as follows.

- Examination of the performance of BERT for the automated detection of actionable reports
- Investigation of the difference in detection performance upon adding order information to the input data

## Methods

### Task description

This study was approved by the institutional review board in our hospital, and was conducted in accordance with the Declaration of Helsinki.

We define two collective terms: (1) “report body,^1^” referring to the findings and impression in radiology reports, and (2) “order information,” referring to the free texts that are written in the ordering form by the referring clinician (e.g., the suspected diseases or indications), as explained in Introduction. Our task is thus defined as the detection of radiology reports with *actionable* tags using the report body alone, or both the order information and the report body.

### Clinical data

We obtained 93,215 confirmed radiology reports for computed tomography (CT) examinations performed at our hospital between September 9, 2019, and April 30, 2021, all of which were written in Japanese. Next, we removed the following radiology reports that were not applicable for this study: (1) eight radiology reports whose findings and impressions were both registered as empty, (2) 254 reports for CT-guided biopsies, and (3) 2030 reports for CT scans for radiation therapy planning. The remaining 90,923 radiology reports corresponded to 18,388 brain, head, and neck; 64,522 body; 522 cardiac; and 5673 musculoskeletal reports; and 3209 reports of other CT examinations whose body parts could not be determined from the information stored in the Radiology Information System (RIS) server. The total was greater than the number of reports because some reports mentioned more than one part.

### Class labeling and data split

Each of the 90,923 radiology reports was defined as actionable (positive class) if it had been provided with an *actionable* tag by the diagnosing radiologist, and it was otherwise defined as non-actionable (negative class). In other words, the gold standard had already been given to all of the reports in the clinical practice, which enabled a fully supervised document classification without additional annotations.

The radiologists in our hospital are requested to regard image findings as actionable when the findings were not supposed to be expected by the referring clinician and were potentially critical if left overlooked. Specific criteria for *actionable* tagging were not determined clearly in advance but left to clinical decisions of individual radiologists.

The numbers of actionable and non-actionable reports were 788 (0.87%) and 90,135 (99.13%), respectively. Then, these radiology reports were split randomly into a training set and a test set in the ratio of 7:3, maintaining the same proportions of actionable and non-actionable reports in each set, i.e., in the training set, there were 63,646 reports, where 552 were actionable and 63,094 were non-actionable, and in the test set, there were 27,277 reports, where 236 were actionable and 27,041 were non-actionable.

### Preprocessing of radiology reports

**Fig. 1:**
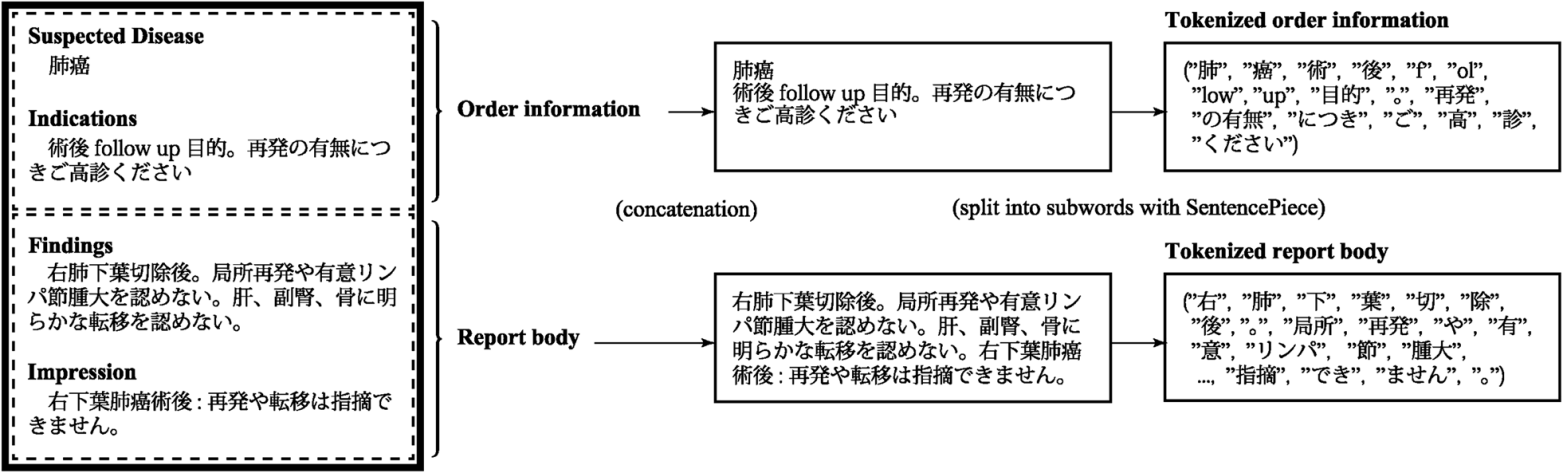
Preprocessing for each radiology report. Note that subwords recognized by SentencePiece are not precisely substrings of words in the grammatical sense, because SentencePiece automatically constructs its vocabulary without a dictionary. For this reason, SentencePiece sometimes treats long phrases as one subword or conversely one character as one subword

To apply machine learning methods in the following sections, the same preprocessing was carried out on all radiology reports (Fig. 1). First, the contents in the order information and report body were respectively concatenated into passages. Then, the passages were individually tokenized with the SentencePiece model, whose vocabulary size is 32,000 [33, 34].

### BERT

BERT is one of the Transformer networks [27, 28]. In general, “Transformer” refers to neural networks using multiple identical encoder or decoder layers with an attention mechanism [35]. Transformer networks have outperformed previous convolutional and recurrent neural networks in NLP tasks [27]. BERT has been proposed as a versatile Transformer network. BERT takes one or two documents as input, passes them into the inner stack of multiple Transformer encoder layers, and characteristically outputs both document-level and token-level representations. BERT can thus be applied to both document-level and token-level classification tasks [28]. Various BERT models pre-trained with large corpora are publicly available, which has established a new ecosystem for pre-training and fine-tuning of NLP models.

We used the Japanese BERT model developed by Kikuta [34]. This model is equivalent to “BERT-base” with 12 Transformer encoder layers and 768-dimensional hidden states. The model has been pre-trained using a Japanese Wikipedia corpus tokenized with the SentencePiece tokenizer [33].

We constructed a binary classifier (hereafter, a BERT classifier) by adding a single-layer perceptron with softmax activation after the pre-trained BERT model. The perceptron converts a 768-dimensional document-level representation vector output by the pre-trained BERT model into a two-dimensional vector.

**Fig. 2:**
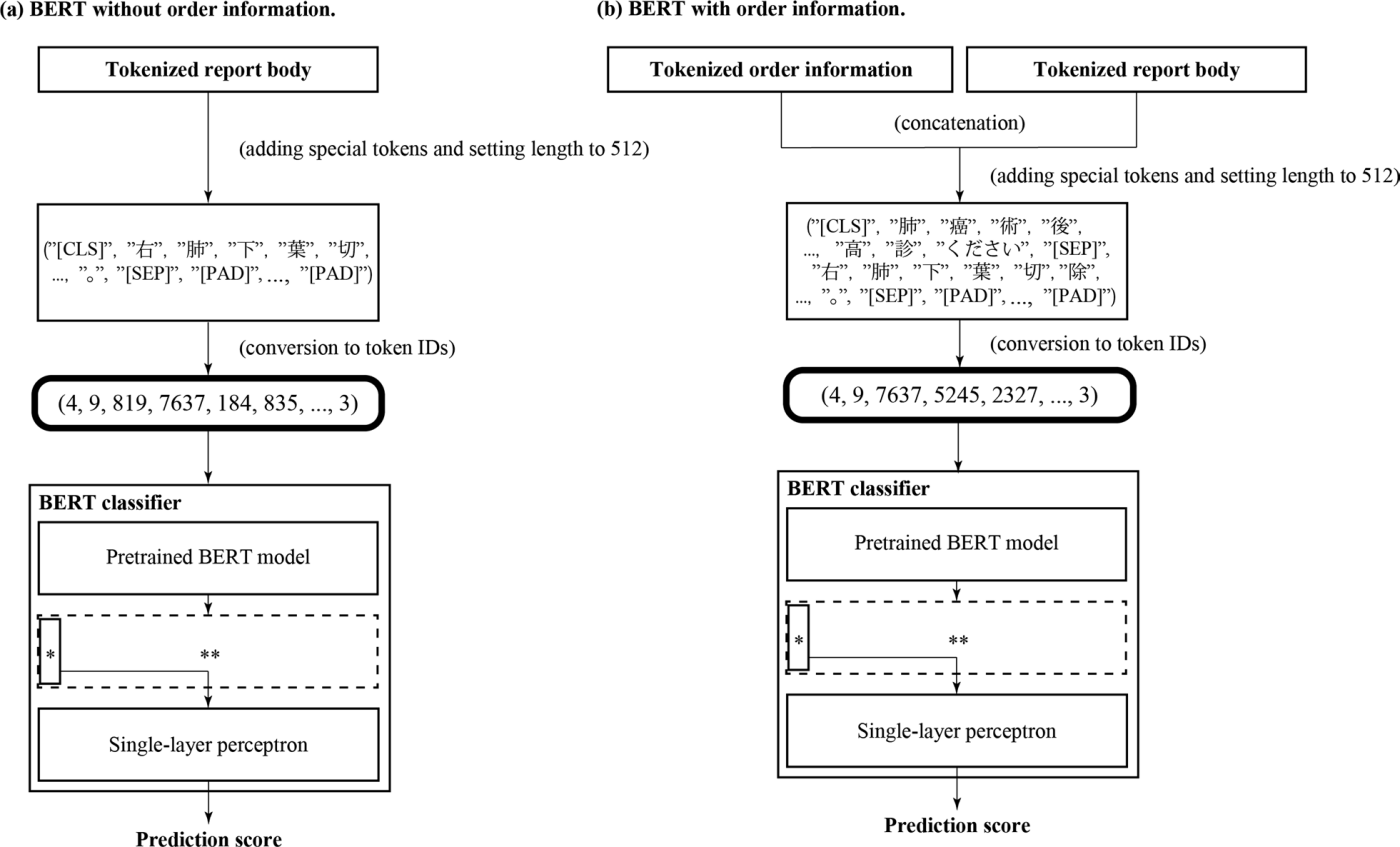
Detection of actionable reports with BERT (**a**) without and (**b**) with order information. Each sequence was fed into the BERT classifier after adding special tokens (e.g., [CLS] and [SEP]) and padding with [PAD] tokens that are required by the standard specification of BERT. Generally, BERT models output a 512 × 768 hidden state matrix (indicated by **), part of which is a 768-dimensional feature vector for classification tasks (indicated by *). We used only the feature vector for classification tasks and discarded the rest of the hidden state matrix, as in the standard procedure

The procedure is shown in Fig. 2. For the detection experiment without order information, the sequences generated from the report body were fed to the BERT classifier. For the detection experiment with order information, each sequence pair generated from the order information and report body was fed to the BERT classifier.

Fine-tuning was performed on all embedding and Transformer encoder layers of the BERT model, and none of these layers were frozen. The maximum sequence length was set to 512 and the batch size^2^ was set to 256. We used Adam optimizer [36] and binary cross-entropy loss function.

As in Table 2, the learning rate and the number of training epochs were set as follows. The learning rate was set to 5.0 × 10^−5^ for the experiment without order information and to 4.0 × 10^−5^ for the experiment with order information. The number of training epochs was set to 3 for both experiments. The learning rate and the number of training epochs were determined by the grid search and five-fold cross-validation using the training set. We tried all of the 25 direct groups of five learning rates, 1.0 × 10^−5^, 2.0 × 10^−5^, 3.0 × 10^−5^, 4.0 × 10^−5^, and 5.0 × 10^−5^, and the five training epochs, 1 to 5. We calculated the averages of the area under the precision-recall curve (AUPRC) [37, 38] for the five folds, and chose the learning rate and the number of training epochs that gave the highest average AUPRC.

**Table 2:** Details of hyperparameter tuning for each method. *x*e+*y* means *x* × 10^*y*^ and *x*e−*y* means *x* × 10^−*y*^

Method	Hyper-parameter	Candidates	Used hyperparameters			
			Order information (−)		Order information (+)	
			Oversampling (−)	Oversampling (+)	Oversampling (−)	Oversampling (+)
LR	C	1e− 4, 1e −3, 1e −2, 1e −1, 1.0, 1e+1, 1e+2, 1e+3, 1e+4	1e+2	1e −2	1.0	1.0
	L1 ratio	0.0, 0.5, 1.0	1.0	0.5	1.0	1.0
GBDT	Iterations	500, 1,000, 1,500	500	1,000	1,500	1,000
LSTM	Learning rate	5e −6, 1e −5, 2e −5, 3e −5	5e −6	1e −5	5e −6	5e −6
	Epochs	5, 10, 15, 20, 25, 30	20	5	20	25
BERT	Learning rate	1e −5, 2e −5, 3e −5, 4e −5, 5e −5	5e −5	5e −5	4e −5	1e −5
	Epochs	1, 2, 3, 4, 5	3	1	3	1

The learning environment was as follows: AMD EPYC 7742 64-Core Processor, 2.0 TB memory, Ubuntu 20.04.2 LTS, NVIDIA A100-SXM4 graphics processing unit (GPU) with 40 GB memory × 6, Python 3.8.10, PyTorch 1.8.1, Torchtext 0.6.0, AllenNLP 2.5.0, PyTorch-Lightning 0.7.6, scikit-learn 0.22.2.post1, Transformers 4.6.1, Tokenizers 0.10.3, SentencePiece 0.1.95, MLflow 1.17.0, and Hydra 0.11.3.

### Baselines: LSTM

As one of the baselines against BERT, we performed automated detections of actionable reports using a two-layer bidirectional long short-term memory (LSTM) model followed by a self-attention layer [27, 39]. As in BERT, the inputs to the LSTM model were report bodies in the experiments without order information and were concatenations of order information and report bodies in the experiments with order information. The lengths of the input documents in a batch were aligned to the longest one by adding special padding tokens at the end of the other documents in the same batch. Next, each document was tokenized and converted into sequences of vocabulary IDs using the SentencePiece tokenizer, and was then passed into a 768-dimensional embedding layer. In short, the preprocessing converted radiology reports in a batch into a batch size × length × 768 tensor.

The final layer of the LSTM model outputs two batch size × length × 768 tensors corresponding to the forward and backward hidden states. We obtained document-level representations by concatenating the two hidden states. The representations were further passed into a single-head self-attention layer with the same architecture as proposed by Vaswani et al. [27]. The self-attention layer converts the document-level representations to a batch size × 1536 matrix by taking the weighted sum of the document-level representations along the time dimension effectively by considering the importance of each token. Then, the matrix was converted into two-dimensional vectors using a single-layer perceptron with softmax activation. The resulting two-dimensional vectors were used as prediction scores. Hereafter, we collectively refer to the LSTM model, the self-attention layer, and the perceptron as the “LSTM classifier.”

We trained the LSTM classifier from scratch. The same optimizer and loss function as those in BERT were used. The batch size was set to 256. As in BERT, the learning rate and the number of training epochs were determined by grid search and five-fold cross-validation. Table 2 shows the hyperparameter candidates on which the grid search was performed and the hyperparameters that were finally chosen for each experiment.

### Baselines: statistical machine learning

Logistic regression (LR) [40] and the gradient boosting decision tree (GBDT) [41] were also examined for comparison.

**Fig. 3:**
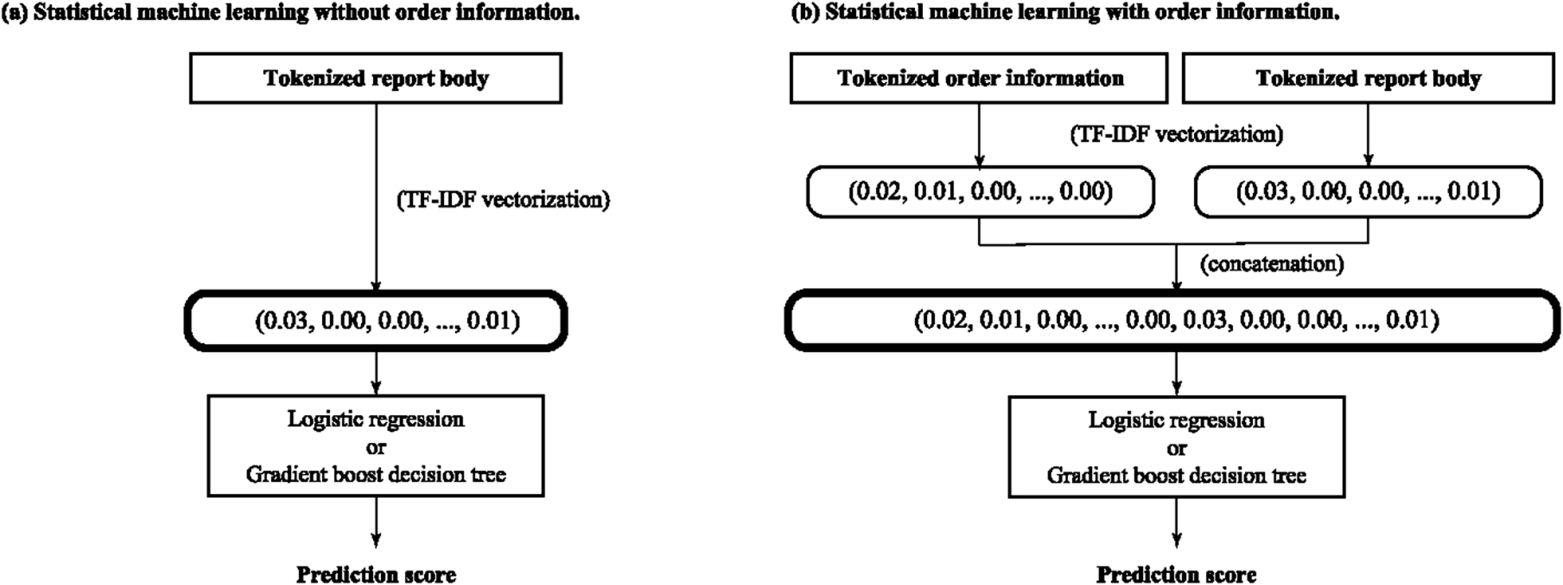
Detection of actionable reports with statistical machine learning (**a**) without and (**b**) with order information

Figure 3 shows the procedures. The tokenized report body and order information were individually converted into term frequency-inverse document frequency (TF-IDF)-transformed count vectors of uni-, bi-, and trigrams (one, two, and three consecutive subwords). The two vectors were concatenated for the detection experiment with order information, and only the vector from the report body was used for the detection experiment without order information.

Here, we describe the details of hyperparameters of the LR and GBDT models. For LR, we used Elastic-Net regularization [30, 42], which regulates model weights with the mixture of L1- and L2-norm regularizations. Elastic-Net takes two parameters, C and the L1 ratio. C is the reciprocal strength to regularize the model weights, and the L1 ratio is the degree of dominance of L1-norm regularization. The C and the L1 ratio were determined with the grid search and five-fold cross-validation, whose candidates and choices are shown in Table 2. For GBDT, the tree depth was set to 6. The number of iterations was determined by grid search and five-fold cross-validation in the same way as LR.

We used the scikit-learn 0.22.2post1 implementation for LR and the CatBoost 0.25.1 [43] implementation for GBDT.

### Performance evaluation

Since this experiment is under a highly imbalanced setting, the performance of each method was mainly evaluated with the AUPRC [37, 38], along with the average precision score.

We statistically compared the AUPRC and average precision among LR, GBDT, LSTM, and BERT using Welch’s t-test with Bonferroni correction [44]. The bootstrapping approach was applied, where 2000 replicates were made, and 2000 AUPRCs and average precisions were calculated for LR, GBDT, LSTM, and BERT. Using the same approach, we also statistically compared the AUPRC and average precision in the experiments without and with order information for each method.

The area under the receiver operating characteristics (ROC) curve (AUROC) was also calculated [45, 46]. The recall, precision, specificity, and F1 score were also calculated at the optimal cut-off point of the ROC curve. The optimal cut-off point was chosen using the minimum distance between the ROC curve and the upper left corner of the plot.

Scikit-learn 0.22.2.post1 implementation was used for calculation of the evaluation metrics, bootstrapping, and statistical analysis.

For a more detailed analysis, we divided the truly actionable reports in the test set into explicit actionable reports (those with expressions recommending follow-up imaging, further clinical investigations, or treatments) and implicit ones (those without such expressions) by manual review by one radiologist (Y. Nakamura, four years of experience in diagnostic radiology). We also calculated recalls for the mass and non-mass subsets of the truly actionable reports in the test set since some previous studies have focused on actionable reports that point out incidental masses or nodules [15–22]. Each of the reports was included in the mass subset when its actionable findings were determined to involve masses or nodules by manual review, otherwise reports were included in the non-mass subset.

### Oversampling

We mainly used the training set mentioned in the previous section, but its significant class imbalance may affect the performance of the automated detection of actionable reports. Oversampling positive data can be one of the methods to minimize the negative impact of the class imbalance [47].

To examine the effectiveness of oversampling, we additionally performed experiments using the oversampled training set. The oversampled training set was created by resampling each actionable radiology report ten times and each non-actionable radiology report once from the original training set. Hyperparameters for each method (LR, GBDT, LSTM, and BERT) and for each input policy (using and not using order information) were determined using the same strategy as that in the experiments without oversampling. The chosen hyperparameters are shown in Table 2.

Note that we did not oversample the validation datasets during the five-fold cross-validation because we intended to search optimal hyperparameters for the same positive class ratio as the test set.

To examine the effect of oversampling, we statistically compared the AUPRC and average precision obtained without and with oversampling in the same way as aforementioned.

## Results

**Fig. 4:**
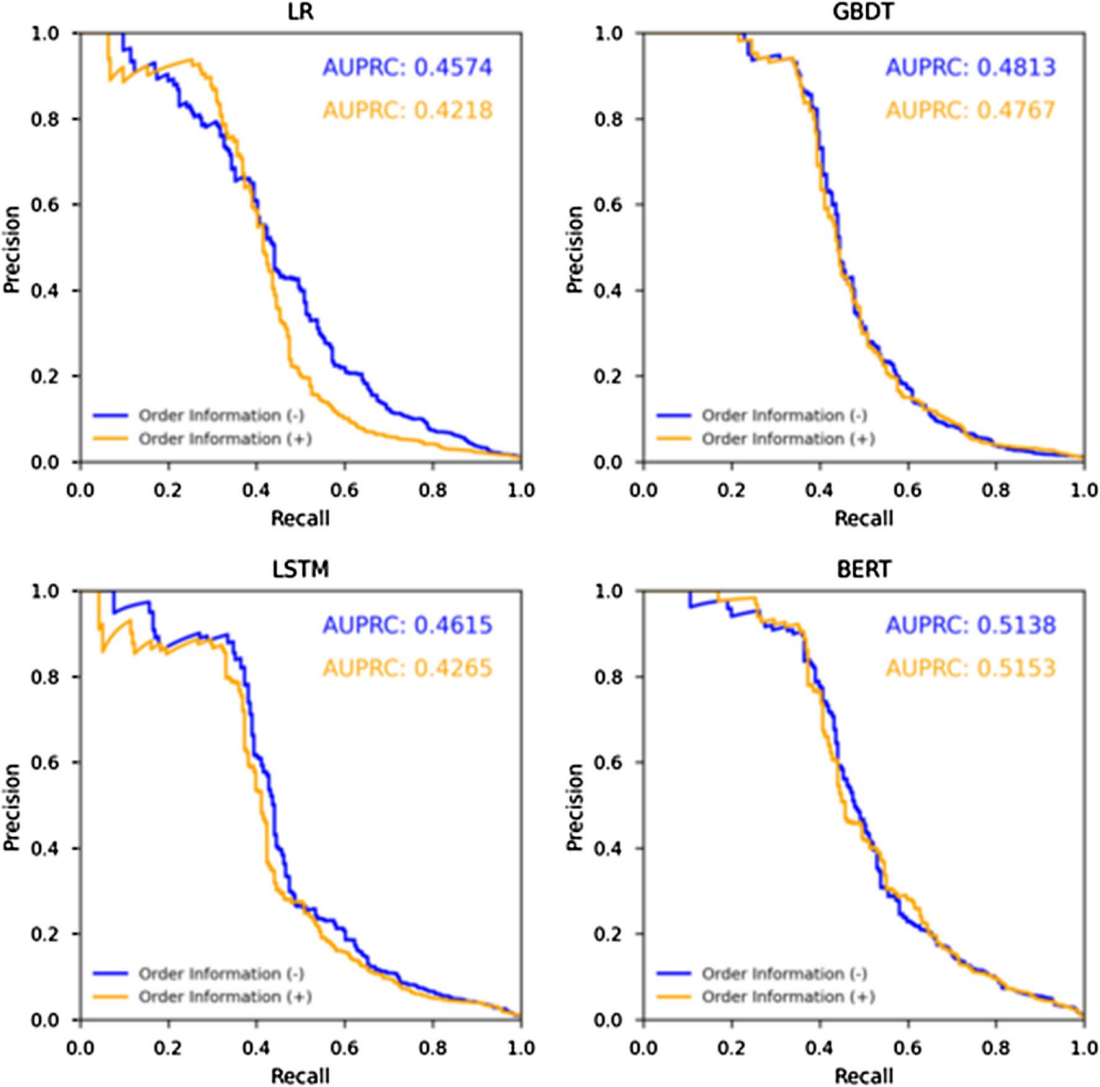
Precision-recall curves for detection of actionable reports achieved by each method

**Fig. 5:**
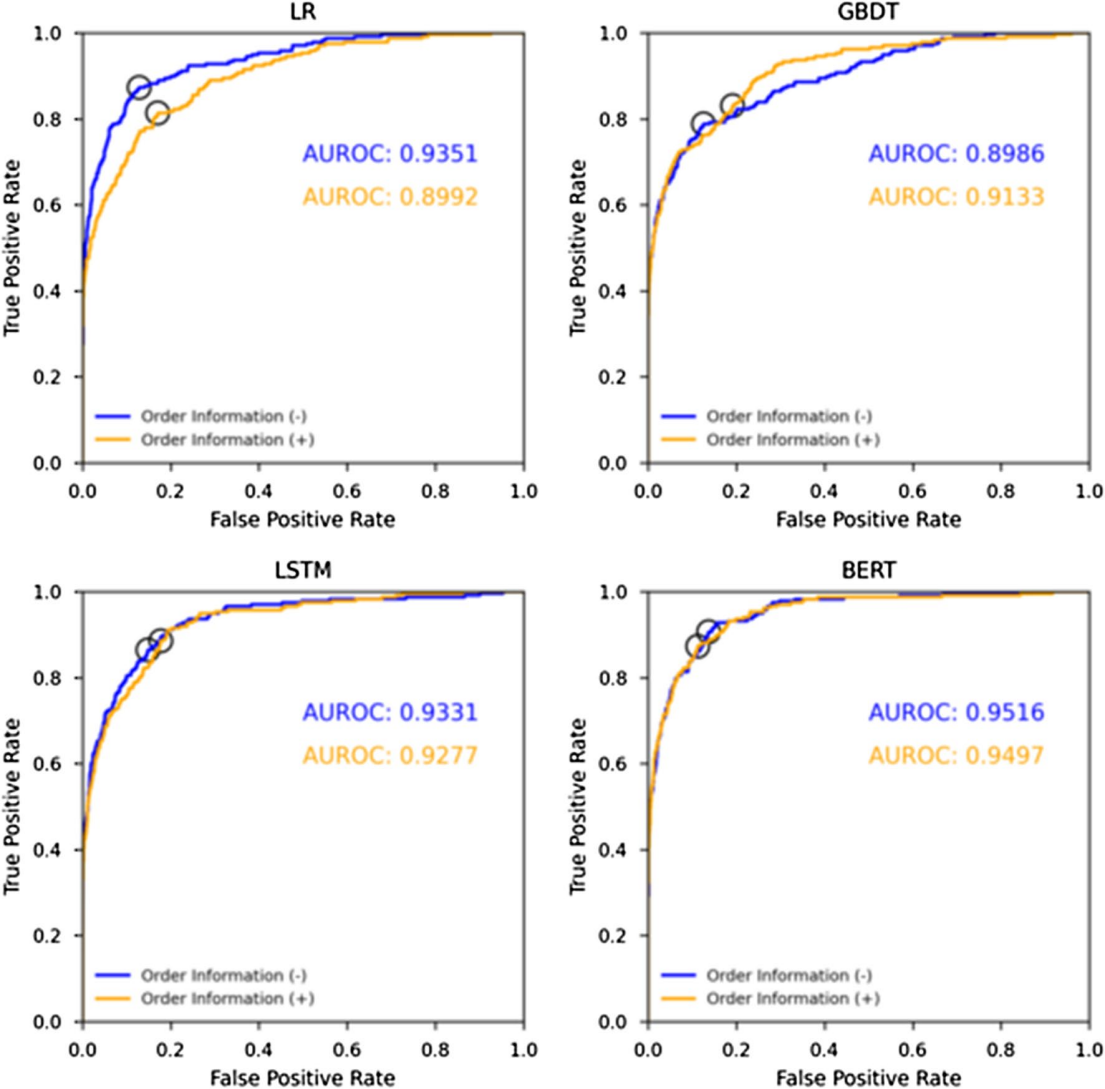
ROC curves for detection of actionable reports achieved by each method, with optimal cut-off points shown as open circles

Figures 4 and 5 show the precision-recall curves and the ROC curves of each method. Table 3 presents the performance of each method calculated from precision-recall curves and optimal cut-off points of ROC curve. Table 4 shows the results of statistical analysis to compare the performance characteristics of LR, GBDT, LSTM, and BERT. In both of the experiments without and with order information, BERT achieved the highest AUPRC and average precision among the four methods, and it showed a statistically significant improvement over the other methods. In particular, the highest AUPRC of 0.5153 was achieved using BERT with order information. The F1 score tended to be higher for the methods with higher AUPRCs, average precisions, and AUROCs. The highest precision was 0.0634, considerably lower than that for recall.

**Table 3:** Performance in detection of actionable reports with different use of order information for each method

Method	Use of order information	AUPRC	Average precision	AUROC	F1 score	Recall	Precision	Specificity
LR	(−)	0.4574	0.4224	0.9351	0.1052	0.8729	0.0559	0.8715
	(+)	0.4218	0.4580	0.8992	0.0763	0.8136	0.0400	0.8296
GBDT	(−)	0.4813	0.4816	0.8986	0.0975	0.7881	0.0520	0.8746
	(+)	0.4767	0.4771	0.9133	0.0699	0.8305	0.0365	0.8087
LSTM	(−)	0.4617	0.4620	0.9331	0.0916	0.8644	0.0484	0.8516
	(+)	0.4265	0.4272	0.9277	0.0797	0.8856	0.0417	0.8226
BERT	(−)	0.5138	0.5142	**0.9516**	0.1030	**0.9068**	0.0546	**0.9271**
	(+)	**0.5153**	**0.5157**	0.9497	**0.1183**	0.8729	**0.0634**	0.9140

Maximum values are shown in bold

**Table 4:** Results of statistical analysis to examine the performance of each detection method

Use of order information	Metrics	Method	*p* values		
			versus GBDT	versus LSTM	versus BERT
(−)	AUPRC	LR	*p* < 0.0001	*p* < 0.0001	*p* < 0.0001
		GBDT	–	*p* < 0.0001	*p* < 0.0001
		LSTM	–	–	*p* < 0.0001
	Average precision	LR	*p* < 0.0001	*p* = 0.0002	*p* < 0.0001
		GBDT	–	*p* < 0.0001	*p* < 0.0001
		LSTM	–	–	*p* < 0.0001
( +)	AUPRC	LR	*p* < 0.0001	*p* < 0.0001	*p* < 0.0001
		GBDT	–	*p* < 0.0001	*p* < 0.0001
		LSTM	–	–	*p* < 0.0001
	Average precision	LR	*p* < 0.0001	*p* < 0.0001	*p* < 0.0001
		GBDT	–	*p* < 0.0001	*p* < 0.0001
		LSTM	–	–	*p* < 0.0001

The advantage of using order information was unclear. Tables 3 and 5 show that the use of order information markedly decreased AUPRC except for BERT. Only BERT slightly improved AUPRC with the use of order information, but the improvement was not statistically significant.

**Table 5:** Results of statistical analysis to examine the impact of use of order information

Method	Metrics	*p* values
		Use of order information ( −) versus ( +)
LR	AUPRC	*p* < 0.0001
	Average precision	*p* < 0.0001
GBDT	AUPRC	*p* < 0.0001
	Average precision	*p* < 0.0001
LSTM	AUPRC	*p* < 0.0001
	Average precision	*p* < 0.0001
BERT	AUPRC	*p* = 0.0972
	Average precision	*p* = 0.1143

Oversampling showed a limited positive effect on the performance. As in Tables 6 and 7, oversampling positive samples in the training dataset ten times resulted in statistically significant improvements of AUPRC and average precision only for GBDT.

**Table 6:** Performance characteristics of methods in detection of actionable reports without and with oversampling of positive samples in the training data

Method	Use of order information	Oversampling	AUPRC	Average precision	AUROC	F1 score
LR	( −)	( −)	**0.4574**	0.4224	**0.9351**	0.1052
		( +)	0.3166	0.3167	0.8036	0.0474
	( +)	( −)	0.4218	**0.4580**	0.8992	0.0763
		( +)	0.4214	0.4221	0.9277	**0.1089**
GBDT	( −)	( −)	0.4813	0.4816	0.8986	0.0975
		( +)	0.4854	0.4858	**0.9335**	0.0841
	( +)	( −)	0.4767	0.4771	0.9133	0.0699
		( +)	**0.4874**	**0.4878**	0.9307	**0.0920**
LSTM	( −)	( −)	**0.4617**	**0.4620**	**0.9331**	**0.0916**
		( +)	0.4188	0.4194	0.9262	0.0818
	( +)	( −)	0.4265	0.4272	0.9277	0.0797
		( +)	0.4086	0.4066	0.9255	0.0795
BERT	( −)	( −)	0.5138	0.5142	**0.9516**	0.1030
		( +)	0.4256	0.4273	0.9464	**0.1190**
	( +)	( −)	**0.5153**	**0.5157**	0.9497	0.1183
		( +)	0.4549	0.4559	0.9441	0.0953

Maximum values for each method are shown in bold

**Table 7:** Results of statistical analysis to examine the impact of oversampling

Method	Use of order information	Metrics	*p* values
			Oversampling ( −) versus ( +)
LR	( −)	AUPRC	*p* < 0.0001
		Average precision	*p* < 0.0001
	( +)	AUPRC	*p* = 0.7971
		Average precision	*p* = 0.9280
GBDT	( −)	AUPRC	*p* = 0.0001
		Average precision	*p* < 0.0001
	( +)	AUPRC	*p* < 0.0001
		Average precision	*p* < 0.0001
LSTM	( −)	AUPRC	*p* < 0.0001
		Average precision	*p* < 0.0001
	( +)	AUPRC	*p* < 0.0001
		Average precision	*p* < 0.0001
BERT	( −)	AUPRC	*p* < 0.0001
		Average Precision	*p* < 0.0001
	( +)	AUPRC	*p* < 0.0001
		Average Precision	*p* < 0.0001

**Fig. 6:**
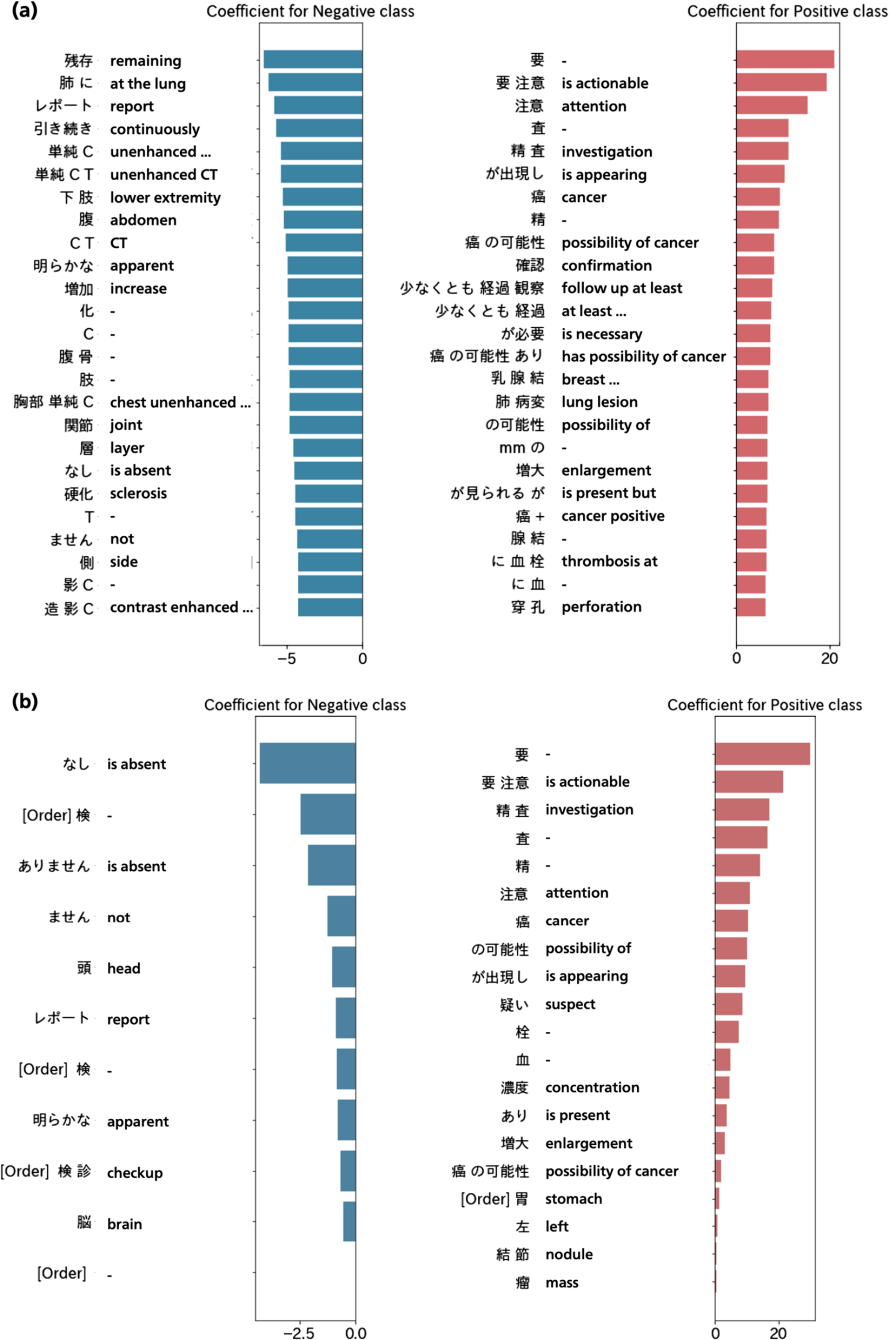
Top *n*-grams with positive and negative coefficients with the largest absolute values of the LR models (**a**) without and (**b**) with order information. Only the top 25 *n*-grams are shown when more than 25 *n*-grams had non-zero coefficients. *N*-grams in order information are marked with [Order]. The translation is not given for *n*-grams too short to make sense. Negation appears among *n*-grams with the smallest negative coefficient

**Fig. 7:**
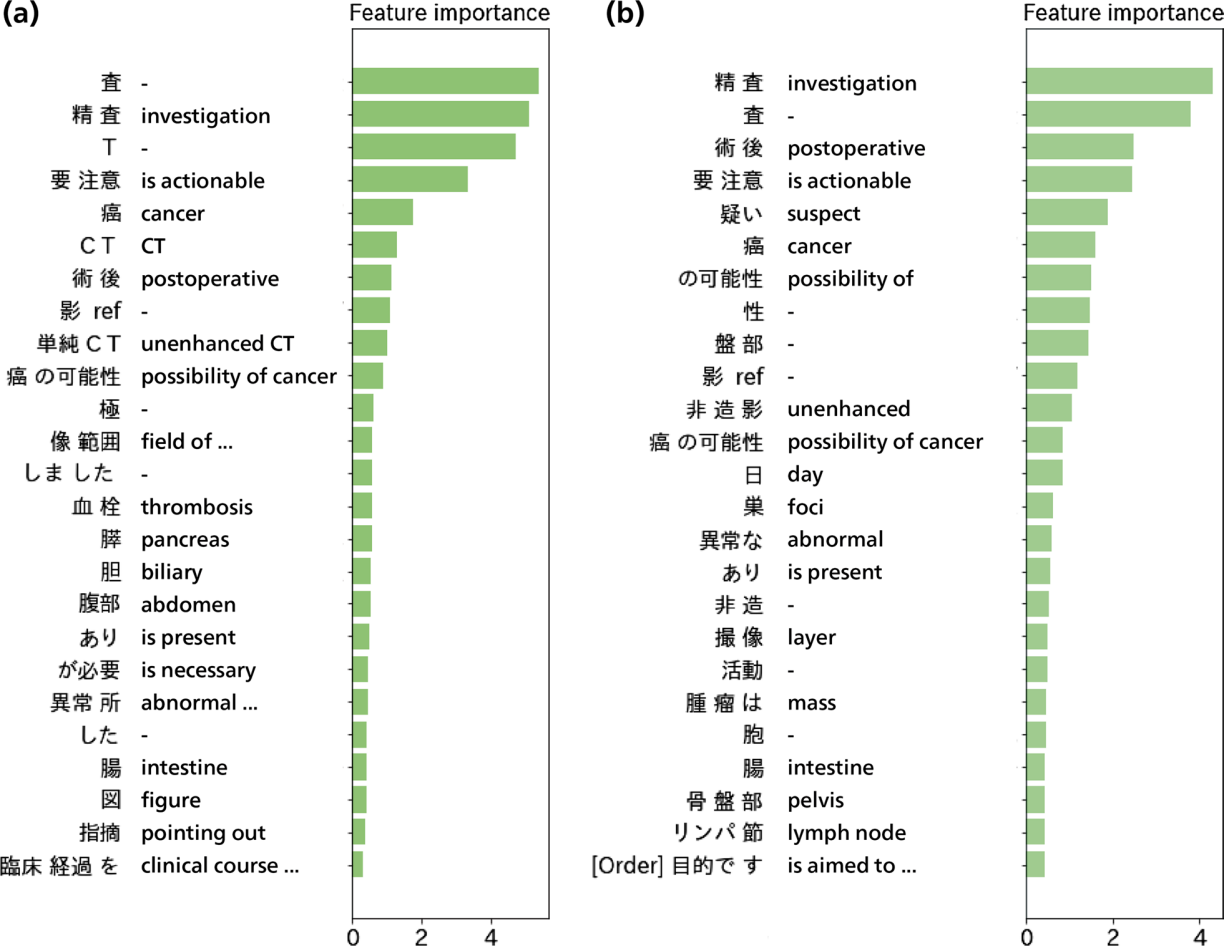
Top 25 *n*-grams with the largest feature importance of GBDT (**a**) without and (**b**) with order information. *N*-grams in order information are marked with [Order]. The translation is not given for *n*-grams too short to make sense

We analyzed further how predictions were made by each method. For LR and GBDT, each of the available *n*-grams (i.e., uni-, bi-, and trigrams) were scored using coefficients assigned by the LR models or feature importance assigned by the GBDT models, which reflected the *n*-grams that the LR and GBDT models placed importance during prediction. *N*-grams consisting only of either Japanese punctuations or Japanese postpositional particles were excluded because they were assumed to be of little value. The results are shown in Figs. 6 and 7, which suggest that the LR and GBDT models tended to predict radiology reports as actionable if they contained such expressions as “is actionable,” “investigation,” “cancer,” or “possibility of cancer.” This suggests that the models picked up explicit remarks by radiologists recommending clinical actions or pointing out cancers. In contrast, patterns in keywords used by the LR model for non-actionable radiology reports were less clear, although some negations such as “is absent” or “not” are observed in Fig. 6b. The word “apparent”, which is frequently accompanied by negative findings in Japanese radiology reporting, is also present in the top negative *n*-grams in Fig. 6b. These imply that the LR model might deduce that radiology reports are non-actionable when negative findings predominate. Order information may not be used much by the LR and GBDT models because few of the *n*-grams in order information are present in Figs. 6 and 7.

**Fig. 8:**
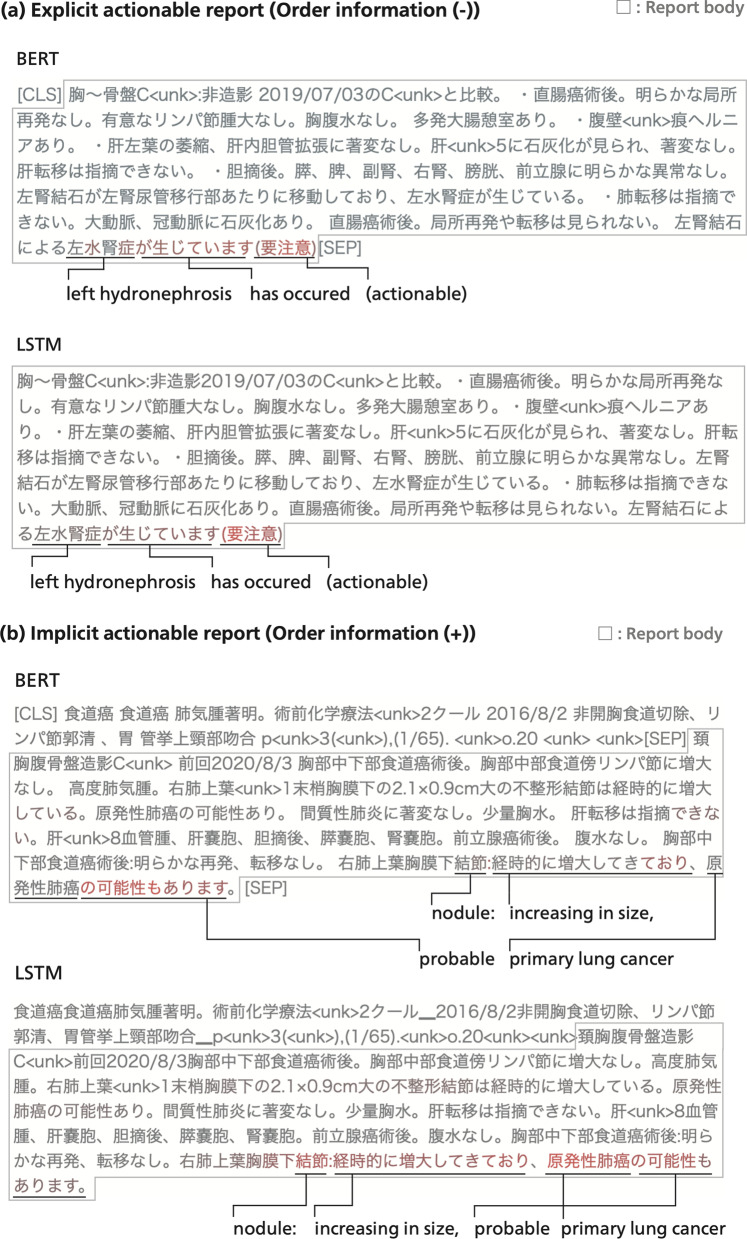
Examples of LSTM and BERT predictions for two truly actionable reports with visualization of attention scores. (**a**) is an explicit actionable report detected without order information, and (**b**) is an implicit actionable report detected using order information. (**a**) Points out hydronephrosis due to ureteral calculus in the postoperative CT examination of rectal cancer, and (**b**) points out a lung nodule pointed out in the CT examination more than four years after the operation of esophageal carcinoma. “ < unk > ” stands for out-of-vocabulary subwords that were not recognized by the LSTM and BERT classifiers. Subwords with relatively high attention scores are colored red. For luminous visualization, Japanese periods are not colored

Figure 8 is a visualization of the self-attention of the LSTM and BERT classifier, highlighting tokens on which large importance was placed by each model during prediction. For LSTM, tokens attracting more attention than others are shown in red. The attention scores were calculated by averaging the row vectors of the attention matrix generated by the self-attention layer. The attention matrix has the length × length size, whose (*i*, *j*) element of the attention matrix stands for the degree of the *i*-th token attending the *j*-th token. Thus, averaging the row vectors can clarify which token is attracting more attention overall than others. For BERT, tokens directing intensive attention toward the [CLS] special token are shown in red. The attention scores were calculated by averaging all of the attention weight matrices in each of the 12 attention heads in the last Transformer encoder layer of the BERT classifier. In Fig. 8, attention scores tended to be higher in expressions such as recommendations or suspicions than in anatomical, radiological, or pathological terms.

Table 8 shows the recalls of each method for the explicit and implicit actionable reports in the test set. 111 truly actionable reports (47%) were implicit in the test set. Although Figs. 6, 7 and 8 imply that all four methods tended to detect actionable findings mainly on the basis of the existence of specific expressions, Table 8 shows that our methods were able to identify actionable reports even if they did not explicitly recommend further medical procedures.

**Fig. 9:**
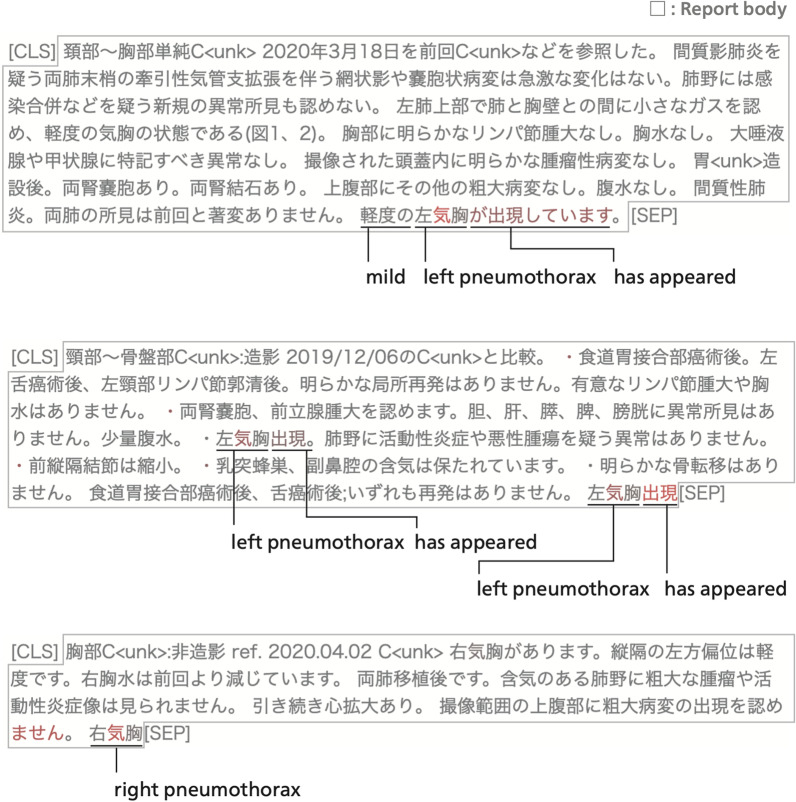
Three implicit actionable radiology reports pointing out incidental pneumothorax, all of which were successfully identified only by BERT. BERT attention scores are visualized in the same way as in Fig. 8. Although none of the three radiology reports emphasize urgency or explicitly recommend clinical actions, BERT has given high attention scores to the disease name “pneumothorax.”

Five of the implicit actionable reports were detected only by BERT and not detected by other methods without order information. Figure 9 shows the BERT attention visualizations towards three of the reports, all of which point out pneumothorax. Although none of the three reports include explicit recommendations or emphatic expressions to highlight actionable findings, BERT successfully predicted them as actionable. Moreover, Figure 9 shows that BERT has assigned high attention scores to a part of the involved disease name “pneumothorax.”

In short, although Figs. 6, 7 and 8 suggest that all four methods mainly relied on whether radiology reports contain specific expressions of recommendation, suspicion, or negation, Fig. 9 implies further the capability of BERT to consider characteristics of diseases.

Table 9 shows the recall for truly actionable reports in the test set. The results in Table 9 suggest that our methods detected actionable reports regardless of the pathological entity of their actionable findings.

As in Table 10, actionable reports accounted for 0.41% of brain, head, and neck; 1.1% of body; and 0.51% of musculoskeletal CT radiology reports in the test set. Table 10 also shows that the recall scores for the actionable musculoskeletal CT reports were greater than those for brain, head, and neck CT reports.

## Discussion

The results show that our method based on BERT outperformed other deep learning methods and statistical machine learning methods in distinguishing various actionable radiology reports from non-actionable ones. The statistical machine learning methods used only limited features, because the radiology reports were converted into the vectors of the frequency of words as the standard feature extraction method [40]. In contrast, BERT and LSTM presumably captured various features of each radiology report including the word order, lexical and syntactic information, and context [28, 29]. Moreover, the superiority of BERT over LSTM was probably brought about by leveraging knowledge from a large amount of pre-training data.

As in Tables 8 and 9, our BERT-based approach was effective in identifying actionable reports regardless of the explicitness or the targeted abnormality. The probable reasons were that (1) implicit actionable reports often emphasized the abnormality that was considered actionable (e.g., “highly suspected to be primary lung cancer” for lung nodules) and that (2) the BERT classifiers were alert to such emphatic expressions in addition to explicit recommendations for follow-up, investigations, or treatment. Furthermore, Figure 9 shows that BERT could still identify implicit actionable reports without emphatic expressions for the actionable findings, and it could assign high attention scores to the names of the actionable findings. This implies that BERT is capable of learning to distinguish disease names that are likely to be often reported as actionable findings.

**Table 8:** Recall scores for explicit and implicit truly actionable reports in the test set

Method	LR		GBDT		LSTM		BERT	
Use of order information	( −)	( +)	( −)	( +)	( −)	( +)	( −)	( +)
Explicit actionable reports (n = 125)	0.960	0.848	0.872	0.912	0.920	0.936	**0.968**	0.928
Implicit actionable reports (n = 111)	0.766	0.766	0.685	0.730	0.793	0.820	**0.829**	0.802

The maximum score for each subset is shown in bold

**Table 9:** Recall scores for truly actionable reports pointing out mass and non-mass abnormalities in the test set

Method	LR		GBDT		LSTM		BERT	
Use of order information	( −)	( +)	( −)	( +)	( −)	( +)	( −)	( +)
Mass subset (n = 124)	**0.935**	0.895	0.839	0.855	0.895	0.927	0.927	0.903
Non-mass subset (n = 112) †	0.795	0.714	0.723	0.795	0.821	0.830	**0.875**	0.830

The maximum value for each subset is shown in bold

^†^Vascular lesions (hemorrhage, thrombosis, infarction, and others) (n = 46), pneumonia (n = 17), pneumothorax (n = 11), hydronephrosis (n = 8), gastrointestinal perforation (n = 4), mediastinal emphysema (n = 3), hydrocephalus (n = 2), and other abnormalities (n = 21)

**Table 10:** Recall for truly actionable reports in the test set calculated for each body part

Body part	#Actionable reports			Recall							
	Total	Implicit	Non-mass	Order information (−)				Order information (+)			
				LR	GBDT	LSTM	BERT	LR	GBDT	LSTM	BERT
Brain, head and neck	23/5584 (0.41%)	10/23 (43.5%)	16/23 (69.6%)	0.739	0.609	**0.870**	**0.870**	0.652	0.783	0.826	0.696
Body	206/19,256 (1.1%)	101/206 (49.0%)	91/206 (44.2%)	0.879	0.801	0.854	**0.903**	0.835	0.825	0.883	0.888
Cardiac	0/151 (0%)	–	–	–	–	–	–	–	–	–	–
Skeletal	9/1758 (0.51%)	1/9 (11.1%)	6/9 (66.7%)	**1.000**	0.889	**1.000**	**1.000**	0.667	**1.000**	**1.000**	0.889
Other	0/959 (0%)	–	–	–	–	–	–	–	–	–	–

Maximum values for each body part are shown in bold

As in Table 10, the detection performance was affected by the body part of the radiology reports. This is probably caused by the difference in the proportion of explicit and mass actionable reports for each body part. The actionable musculoskeletal CT reports were more often explicit and targeting mass abnormality than the brain, head, and neck CT reports. Tables 8 and 9 suggest that explicit and mass actionable reports were comparatively easier to identify than implicit and non-mass ones. This was probably why all four methods achieved higher recalls scores for musculoskeletal actionable reports than brain, head, and neck ones.

Order information did not necessarily improve the performance. This may be because the truly actionable reports had a too diverse relationship between the order information and the report body. We found that the *actionable* tags were not only used to caution about findings that were irrelevant to the main purpose of ordering (e.g., lung nodules found in a CT examination to diagnose fracture). Rather, the *actionable* tags were also given to the radiology reports to highlight unusual clinical courses (e.g., liver metastases from colon cancer first appeared five years after the surgery of the primary lesion) or to prompt immediate treatments (e.g., hemorrhage in the nasal septum associated with nasal fracture). These complex situations may have not been recognized well from our small dataset, even with the ability of BERT to capture the relationship between the report body and order information.

The low precision (0.0365–0.0634) was another problem in this study. It was probably mainly due to the low positive case ratio (0.87%). Generally, an imbalance of occurrences between positive and negative samples strongly hampers a binary classification task [48]. This negative impact of low positive case ratio was not alleviated by simple oversampling, probably because it did not provide bring new information to learn characteristics of actionable reports to the models. To overcome this limitation, obtaining a larger amount of positive data by collecting more radiology reports or data augmentation [49] may be an effective solution. Other approaches such as cost-sensitive learning [50] or the use of dice loss function [51] can also be worth trying in future studies.

An important advantage of the proposed approach in this study is that the radiology reports were labeled with tags provided in actual radiological practice. Generally, radiologists determine whether specific findings are actionable or not on the basis of not only radiological imaging but also a comparison with a prior series of images, order information, and electronic health records. The actionable tag can consequently reflect such clinical decisions. Therefore, there is probably room for improvement in the performance of automated detection of actionable reports by using the imaging data themselves and the information in electronic health records. This benefit may not be obtained by independent class labeling, referring only to the sentences in the radiology reports.

Using the *actionable* tag as the label has another merit: to identify implicit actionable reports. The results of this study suggest that the radiologists may have sometimes thought that actionable findings were present in the radiological images without explicitly urging further clinical examinations or treatments in the radiology report. The labeling and detection methods in this study identified such implicit actionable reports, though with lower performance than those for explicit ones.

Another advantage of the approach of this study is that actionable findings for any pathological entity were dealt with, thereby realizing comprehensive detection. Since various diseases appear as actionable findings in radiological imaging [1, 7–15], this wide coverage is considered essential for better clinical practice.

The *actionable* tagging itself can play a certain role in the clinical management of actionable reports. Nonetheless, introducing an automated detection system for actionable findings can make further contributions by providing decisions complementary to those of the radiologists. This is because different radiologists have been shown to act differently to actionable findings [52], and there have been no specific criteria for actionable tagging in our hospital thus far.

There are several limitations of the approach of this study. First, the BERT model used in this study was not specialized in the biomedical domain. The BERT model failed to recognize about 1% of the words, most of which were abbreviations or uncommon Chinese characters of medical terms. Kawazoe et al. have recently provided a BERT model pre-trained with Japanese clinical records, which may improve the performance [53]. The pre-training of BERT with a large Japanese biomedical corpus is worthwhile as future work, although it can be costly from the viewpoint of computational resources. Second, the short period since the launch of *actionable* tagging in our hospital meant that the amount of data was limited. Continuous *actionable* tagging operations can lead to larger datasets. Finally, since this study is a single-institution study, our classifiers may be adapted to the epidemiology, the style of reporting, and the principle on actionable findings unique to our hospital. Expanding this study to other institutions with similar systems of reporting and communication will be valuable future work.

## Conclusions

We have investigated the automated detection of radiology reports with actionable findings using BERT. The results showed that our method based on BERT is more useful for distinguishing various actionable radiology reports from non-actionable ones than models based on other deep learning methods or statistical machine learning.

## Abbreviations

AUPRC: Area under the precision-recall curve
AUROC: Area under the receiver operating characteristics curve
BERT: Bidirectional encoder representations from transformers
CNN: Convolutional neural network
GRU: Gated recurrent units
CT: Computed tomography
GBDT: Gradient boosting decision tree
LR: Logistic regression
LSTM: Long short-term memory
NLP: Natural language processing
RIS: Radiology information system
ROC: Receiver operating characteristics
SML: Statistical machine learning
TF-IDF: Term frequency-inverse document frequency

## Footnotes

1 For simplicity, we regarded impression as part of the report body, although this is different from the definition of the body of the report by the American College of Radiology [32].

2 The actual batch size was set to 16 owing to the limited computational resources. However, an effective batch size of 256 was realized by accumulating gradients of every 16 batches with the PyTorch-Lightning implementation.

## Declarations

### Availability of data and materials

The radiology reports and order information involved in this study are not publicly available because publishing the dataset is not approved by the institutional review board of the University of Tokyo Hospital. For more information, please contact the corresponding authors.
